# Methyl 4-hy­droxy-2-meth­oxy­carbonyl­methyl-1,1-dioxo-1,2-dihydro-1λ^6^,2-benzothia­zine-3-carboxyl­ate^1^

**DOI:** 10.1107/S1600536811034921

**Published:** 2011-08-31

**Authors:** Muhammad Nadeem Arshad, Islam Ullah Khan, Muhammad Zia-ur-Rehman, Sheikh Asrar Ahmad, H. M. Rafique

**Affiliations:** a X-ray Diffraction and Crystallography Laboratory, Department of Physics, School of Physical Sciences, University of the Punjab, Quaid-e-Azam Campus, Lahore 54590, Pakistan; b Materials Chemistry Laboratory, Department of Chemistry, GC University, Lahore 54000, Pakistan; c Applied Chemistry Research Centre, PCSIR Laboratories Complex, Lahore 54600, Pakistan; d Department of Chemistry, Division of Science and Technology, University of Education, Lahore, Pakistan

## Abstract

There are two independent mol­ecules in the asymmetric unit of the title compound, C_13_H_13_NO_7_S, which have almost identical geometries. The thia­zine ring adopts a sofa conformation in both mol­ecules and the mol­ecular conformations are stabilized by intramolecular O—H⋯O hydrogen bonds. Inter­molecular C—H⋯O hydrogen bonds stabilize the crystal packing.

## Related literature

For related structures, see; Arshad *et al.* (2009 ▶, 2010 ▶). For the synthesis, see; Arshad *et al.* (2011 ▶).
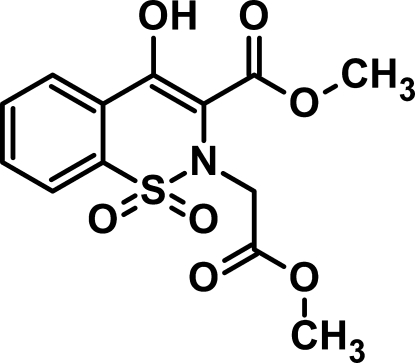


## Experimental

### 

#### Crystal data

C_13_H_13_NO_7_S
*M* *_r_* = 327.30
Triclinic, 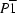

*a* = 8.9128 (14) Å
*b* = 12.414 (2) Å
*c* = 13.443 (2) Å
α = 79.784 (2)°
β = 72.981 (3)°
γ = 88.503 (3)°
*V* = 1399.2 (4) Å^3^
*Z* = 4
Mo *K*α radiation
μ = 0.27 mm^−1^
*T* = 100 K
0.48 × 0.36 × 0.33 mm

#### Data collection

Siemens SMART diffractometer equipped with a Bruker APEXII detector
Absorption correction: multi-scan (*SADABS*; Bruker, 2001 ▶) *T*_min_ = 0.882, *T*_max_ = 0.917
17331 measured reflections
6923 independent reflections
6322 reflections with *I* > 2σ(*I*)
*R*_int_ = 0.018

#### Refinement

*R*[*F* ^2^ > 2σ(*F* ^2^)] = 0.040
*wR*(*F* ^2^) = 0.107
*S* = 1.06
6923 reflections
407 parameters
H atoms treated by a mixture of independent and constrained refinement
Δρ_max_ = 0.48 e Å^−3^
Δρ_min_ = −0.42 e Å^−3^

### 

Data collection: *APEX2* (Bruker, 2001 ▶); cell refinement: *SAINT* (Bruker, 2001 ▶); data reduction: *SAINT*; program(s) used to solve structure: *SHELXS97* (Sheldrick, 2008 ▶); program(s) used to refine structure: *SHELXL97* (Sheldrick, 2008 ▶); molecular graphics: *PLATON* (Spek, 2009 ▶) and *X-SEED* (Barbour, 2001 ▶); software used to prepare material for publication: *WinGX* (Farrugia, 1999 ▶).

## Supplementary Material

Crystal structure: contains datablock(s) I, global. DOI: 10.1107/S1600536811034921/bt5629sup1.cif

Structure factors: contains datablock(s) I. DOI: 10.1107/S1600536811034921/bt5629Isup2.hkl

Supplementary material file. DOI: 10.1107/S1600536811034921/bt5629Isup3.cml

Additional supplementary materials:  crystallographic information; 3D view; checkCIF report

## Figures and Tables

**Table 1:** Hydrogen-bond geometry (Å, °)

*D*—H⋯*A*	*D*—H	H⋯*A*	*D*⋯*A*	*D*—H⋯*A*
O1—H1*O*⋯O4	0.79 (3)	1.87 (3)	2.5754 (18)	149 (3)
O8—H8*O*⋯O11	0.98 (3)	1.68 (3)	2.5623 (17)	148 (3)
C13—H13*A*⋯O10^i^	0.98	2.44	3.202 (2)	134
C2—H2⋯O11^ii^	0.95	2.59	3.339 (2)	136

Symmetry codes: (i) 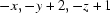; (ii) 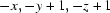.

**Fig. 1.**
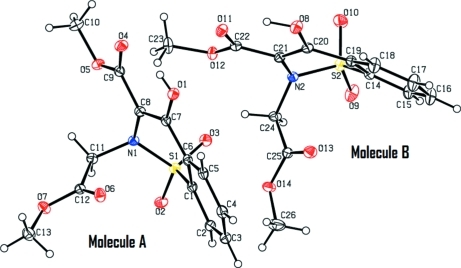
The ORTEP diagram of the title compound with displacement ellipsoids drawn at the 30% probability level.

**Fig. 2.**
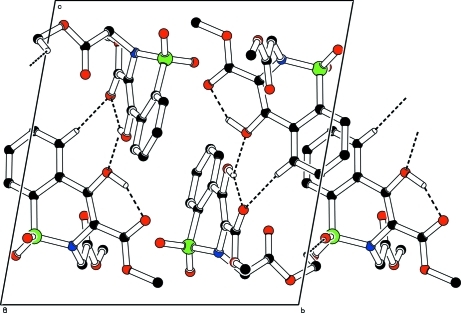
Unit cell packing of the title compound showing hydrogen bonding interaction as dashed lines.

## supplementary crystallographic information

### Comment

The crystal structure of title compound is being reported in continuation of our structural studies of various benzothiazines (Arshad *et al.*, 2009, 2010).

The title compound   crystallizes with two independent molecules in the asymmetric  unit (Fig. 1). In molecule A, the two fused rings are oriented at dihedral angle of 14.18 (7)° while in B this dihedral angle amounts to 18.40 (6)°. The root mean square deviation values for thiazine ring in both molecules are 0.1991Å and 0.2119Å respectively. The alkylated groups attached to the nitrogen atom in molecule A and B are inclined at almost perpendicular position with respect to the thiazine rings and given values are 85.81 (5)° and 88.12 (5)°.  Both of the molecules are involved in intra and intermolecular hydrogen bondings. The intramolecular hydrogen bonding of O—H···O type forms six membered ring motif in each molecule. Other inter molecular C—H···O and O—H···O type hydrogen bonding interactions produce three dimensional network (Fig. 2 and Tab. 1).

### Experimental

The synthesis of title compound is already reported (Arshad *et al.*, 2011). The compound was recrystallized under slow evaporation technique in ethylacetate.

### Refinement

The C-H H-atoms were positioned with idealized geometry with C—H = 0.95 Å for aromatic, C—H = 0.99 Å for methylene &  C—H = 0.98 Å for methyl groups and were refined using a riding model with *U*_iso_(H) = 1.2 *U*_eq_(C) for aromatic & methylene and *U*_iso_(H) = 1.5 *U*_eq_(C) for methyl groups.

The O—H H atoms were located in a difference map and refined isotropically.

### Crystal data

**Table:**

C_13_H_13_NO_7_S	*Z* = 4
*M**_r_* = 327.30	*F*(000) = 680
Triclinic, *P*1	*D*_x_ = 1.554 Mg m^−^^3^
Hall symbol: -P 1	Mo *K*α radiation, λ = 0.71073 Å
*a* = 8.9128 (14) Å	Cell parameters from 9730 reflections
*b* = 12.414 (2) Å	θ = 2.4–28.9°
*c* = 13.443 (2) Å	µ = 0.27 mm^−^^1^
α = 79.784 (2)°	*T* = 100 K
β = 72.981 (3)°	Blocks, colorless
γ = 88.503 (3)°	0.48 × 0.36 × 0.33 mm
*V* = 1399.2 (4) Å^3^	

### Data collection

**Table:**

Siemens SMART diffractometer equipped with a Bruker APEXII detector	6923 independent reflections
Radiation source: fine-focus sealed tube	6322 reflections with *I* > 2σ(*I*)
graphite	*R*_int_ = 0.018
φ and ω scans	θ_max_ = 28.9°, θ_min_ = 2.1°
Absorption correction: multi-scan (*SADABS*; Bruker, 2001)	*h* = −12→12
*T*_min_ = 0.882, *T*_max_ = 0.917	*k* = −16→16
17331 measured reflections	*l* = −18→17

### Refinement

**Table:**

Refinement on *F*^2^	Primary atom site location: structure-invariant direct methods
Least-squares matrix: full	Secondary atom site location: difference Fourier map
*R*[*F*^2^ > 2σ(*F*^2^)] = 0.040	Hydrogen site location: inferred from neighbouring sites
*wR*(*F*^2^) = 0.107	H atoms treated by a mixture of independent and constrained refinement
*S* = 1.06	*w* = 1/[σ^2^(*F*_o_^2^) + (0.0516*P*)^2^ + 0.9585*P*] where *P* = (*F*_o_^2^ + 2*F*_c_^2^)/3
6923 reflections	(Δ/σ)_max_ = 0.001
407 parameters	Δρ_max_ = 0.48 e Å^−^^3^
0 restraints	Δρ_min_ = −0.42 e Å^−^^3^

### Special details

**Table:**

Geometry. All esds (except the esd in the dihedral angle between two l.s. planes) are estimated using the full covariance matrix. The cell esds are taken into account individually in the estimation of esds in distances, angles and torsion angles; correlations between esds in cell parameters are only used when they are defined by crystal symmetry. An approximate (isotropic) treatment of cell esds is used for estimating esds involving l.s. planes.
Refinement. Refinement of F^2^ against ALL reflections. The weighted R-factor wR and goodness of fit S are based on F^2^, conventional R-factors R are based on F, with F set to zero for negative F^2^. The threshold expression of F^2^ > 2sigma(F^2^) is used only for calculating R-factors(gt) etc. and is not relevant to the choice of reflections for refinement. R-factors based on F^2^ are statistically about twice as large as those based on F, and R- factors based on ALL data will be even larger.

### Fractional atomic coordinates and isotropic or equivalent isotropic displacement parameters (Å^2^)

**Table:**

	*x*	*y*	*z*	*U*_iso_*/*U*_eq_	
S1	0.16821 (4)	0.59653 (3)	0.19082 (3)	0.01849 (9)	
S2	0.52552 (5)	0.92829 (3)	0.77528 (3)	0.02196 (10)	
O8	0.48743 (15)	0.72159 (10)	0.56519 (9)	0.0263 (3)	
O1	0.30206 (15)	0.68027 (10)	0.44663 (9)	0.0240 (2)	
O12	0.38864 (14)	0.59665 (9)	0.88368 (9)	0.0225 (2)	
O4	0.56249 (14)	0.75774 (10)	0.31183 (9)	0.0267 (3)	
O7	0.12303 (14)	0.95735 (10)	0.08091 (9)	0.0269 (3)	
O11	0.39383 (14)	0.56505 (10)	0.72314 (9)	0.0259 (2)	
O14	0.89610 (14)	0.70395 (10)	0.86890 (10)	0.0276 (3)	
O3	0.25344 (14)	0.49769 (9)	0.20325 (9)	0.0245 (2)	
N1	0.29039 (15)	0.70033 (10)	0.17185 (10)	0.0181 (2)	
O13	0.86686 (14)	0.77263 (11)	0.70832 (10)	0.0286 (3)	
O2	0.09291 (14)	0.61619 (10)	0.10870 (9)	0.0236 (2)	
O5	0.59329 (13)	0.76066 (10)	0.13910 (9)	0.0229 (2)	
N2	0.53834 (15)	0.79462 (11)	0.80675 (10)	0.0179 (2)	
O9	0.59840 (17)	0.97841 (11)	0.83798 (10)	0.0343 (3)	
O6	0.06605 (15)	0.85459 (10)	0.24309 (9)	0.0275 (3)	
O10	0.36468 (15)	0.94991 (11)	0.78104 (11)	0.0342 (3)	
C19	0.61858 (19)	0.87842 (13)	0.58043 (12)	0.0211 (3)	
C11	0.27512 (18)	0.80160 (13)	0.10098 (12)	0.0202 (3)	
H11A	0.2567	0.7827	0.0369	0.024*	
H11B	0.3751	0.8446	0.0785	0.024*	
C3	−0.2273 (2)	0.57856 (14)	0.43419 (13)	0.0249 (3)	
H3	−0.3349	0.5583	0.4499	0.030*	
C14	0.63731 (19)	0.95418 (13)	0.64225 (12)	0.0203 (3)	
C9	0.50907 (19)	0.74369 (12)	0.24092 (12)	0.0201 (3)	
C8	0.34682 (18)	0.70707 (12)	0.26034 (12)	0.0189 (3)	
C20	0.53123 (18)	0.77482 (13)	0.63114 (12)	0.0200 (3)	
C6	0.08927 (18)	0.64003 (12)	0.38905 (12)	0.0188 (3)	
C1	0.03184 (18)	0.60509 (12)	0.31319 (12)	0.0187 (3)	
C2	−0.12533 (19)	0.57587 (13)	0.33394 (13)	0.0215 (3)	
H2	−0.1625	0.5545	0.2807	0.026*	
C15	0.7366 (2)	1.04599 (13)	0.60003 (14)	0.0269 (3)	
H15	0.7499	1.0952	0.6436	0.032*	
C5	−0.01566 (19)	0.64217 (12)	0.48944 (12)	0.0216 (3)	
H5	0.0204	0.6651	0.5424	0.026*	
C22	0.42446 (18)	0.62469 (13)	0.77922 (12)	0.0204 (3)	
C24	0.64439 (19)	0.75062 (14)	0.86710 (12)	0.0229 (3)	
H24A	0.6064	0.6760	0.9049	0.027*	
H24B	0.6399	0.7964	0.9211	0.027*	
C21	0.50090 (17)	0.73286 (12)	0.73654 (12)	0.0184 (3)	
C18	0.6976 (2)	0.89909 (15)	0.47193 (13)	0.0302 (4)	
H18	0.6846	0.8501	0.4280	0.036*	
C12	0.14236 (18)	0.87217 (13)	0.15144 (12)	0.0207 (3)	
C25	0.81345 (18)	0.74493 (13)	0.80252 (13)	0.0209 (3)	
C4	−0.1721 (2)	0.61080 (13)	0.51154 (13)	0.0246 (3)	
H4	−0.2422	0.6113	0.5800	0.029*	
C23	0.3242 (2)	0.48585 (14)	0.92729 (14)	0.0303 (4)	
H23A	0.2267	0.4773	0.9093	0.045*	
H23B	0.3021	0.4727	1.0043	0.045*	
H23C	0.4002	0.4330	0.8976	0.045*	
C10	0.7541 (2)	0.79979 (16)	0.11653 (14)	0.0290 (4)	
H10A	0.7569	0.8799	0.1072	0.043*	
H10B	0.8190	0.7768	0.0517	0.043*	
H10C	0.7949	0.7690	0.1755	0.043*	
C7	0.25286 (19)	0.67701 (12)	0.36224 (12)	0.0195 (3)	
C17	0.7950 (3)	0.99176 (17)	0.42903 (14)	0.0366 (4)	
H17	0.8479	1.0059	0.3554	0.044*	
C13	0.0082 (2)	1.03446 (15)	0.12689 (15)	0.0309 (4)	
H13A	−0.0919	0.9955	0.1652	0.046*	
H13B	−0.0070	1.0915	0.0705	0.046*	
H13C	0.0464	1.0681	0.1759	0.046*	
C16	0.8164 (3)	1.06409 (15)	0.49221 (15)	0.0348 (4)	
H16	0.8855	1.1260	0.4618	0.042*	
C26	1.0610 (2)	0.6897 (2)	0.8203 (2)	0.0437 (5)	
H26A	1.0733	0.6389	0.7705	0.066*	
H26B	1.1107	0.6600	0.8750	0.066*	
H26C	1.1108	0.7606	0.7823	0.066*	
H1O	0.389 (4)	0.703 (3)	0.425 (2)	0.066*	
H8O	0.437 (4)	0.653 (3)	0.608 (2)	0.066*	

### Atomic displacement parameters (Å^2^)

**Table:**

	*U*^11^	*U*^22^	*U*^33^	*U*^12^	*U*^13^	*U*^23^
S1	0.01975 (18)	0.01908 (18)	0.01976 (18)	0.00083 (13)	−0.00782 (14)	−0.00819 (13)
S2	0.0236 (2)	0.02065 (19)	0.02086 (19)	0.00131 (14)	−0.00283 (14)	−0.00805 (14)
O8	0.0324 (6)	0.0306 (6)	0.0196 (5)	−0.0077 (5)	−0.0116 (5)	−0.0059 (5)
O1	0.0298 (6)	0.0259 (6)	0.0195 (5)	−0.0054 (5)	−0.0127 (5)	−0.0018 (4)
O12	0.0272 (6)	0.0190 (5)	0.0187 (5)	−0.0014 (4)	−0.0031 (4)	−0.0024 (4)
O4	0.0269 (6)	0.0318 (6)	0.0258 (6)	−0.0035 (5)	−0.0129 (5)	−0.0069 (5)
O7	0.0287 (6)	0.0288 (6)	0.0216 (5)	0.0065 (5)	−0.0076 (5)	−0.0012 (5)
O11	0.0279 (6)	0.0255 (6)	0.0253 (6)	−0.0050 (5)	−0.0079 (5)	−0.0062 (5)
O14	0.0231 (6)	0.0296 (6)	0.0337 (6)	0.0044 (5)	−0.0146 (5)	−0.0047 (5)
O3	0.0268 (6)	0.0202 (5)	0.0302 (6)	0.0040 (4)	−0.0102 (5)	−0.0111 (5)
N1	0.0196 (6)	0.0197 (6)	0.0178 (6)	−0.0005 (5)	−0.0083 (5)	−0.0055 (5)
O13	0.0232 (6)	0.0343 (7)	0.0254 (6)	0.0027 (5)	−0.0037 (5)	−0.0036 (5)
O2	0.0236 (6)	0.0301 (6)	0.0216 (5)	−0.0004 (4)	−0.0102 (4)	−0.0097 (5)
O5	0.0193 (5)	0.0272 (6)	0.0239 (5)	−0.0013 (4)	−0.0064 (4)	−0.0088 (4)
N2	0.0194 (6)	0.0204 (6)	0.0160 (6)	0.0000 (5)	−0.0075 (5)	−0.0048 (5)
O9	0.0453 (8)	0.0330 (7)	0.0241 (6)	−0.0119 (6)	−0.0030 (5)	−0.0142 (5)
O6	0.0313 (6)	0.0231 (6)	0.0235 (6)	0.0025 (5)	−0.0023 (5)	−0.0025 (4)
O10	0.0265 (6)	0.0325 (7)	0.0385 (7)	0.0104 (5)	−0.0024 (5)	−0.0063 (6)
C19	0.0250 (8)	0.0216 (7)	0.0185 (7)	−0.0011 (6)	−0.0098 (6)	−0.0021 (6)
C11	0.0222 (7)	0.0225 (7)	0.0167 (7)	0.0009 (6)	−0.0068 (6)	−0.0039 (5)
C3	0.0215 (8)	0.0227 (8)	0.0288 (8)	0.0004 (6)	−0.0061 (6)	−0.0019 (6)
C14	0.0233 (7)	0.0194 (7)	0.0182 (7)	0.0026 (6)	−0.0065 (6)	−0.0031 (5)
C9	0.0230 (7)	0.0169 (7)	0.0227 (7)	0.0013 (5)	−0.0091 (6)	−0.0054 (5)
C8	0.0222 (7)	0.0186 (7)	0.0196 (7)	0.0008 (5)	−0.0099 (6)	−0.0064 (5)
C20	0.0200 (7)	0.0233 (7)	0.0194 (7)	−0.0008 (6)	−0.0081 (6)	−0.0063 (6)
C6	0.0236 (7)	0.0143 (6)	0.0195 (7)	0.0002 (5)	−0.0079 (6)	−0.0028 (5)
C1	0.0226 (7)	0.0155 (6)	0.0189 (7)	0.0012 (5)	−0.0068 (6)	−0.0044 (5)
C2	0.0228 (7)	0.0187 (7)	0.0251 (7)	0.0000 (6)	−0.0097 (6)	−0.0043 (6)
C15	0.0349 (9)	0.0193 (8)	0.0265 (8)	−0.0023 (6)	−0.0085 (7)	−0.0045 (6)
C5	0.0293 (8)	0.0178 (7)	0.0183 (7)	0.0008 (6)	−0.0080 (6)	−0.0028 (5)
C22	0.0167 (7)	0.0230 (7)	0.0216 (7)	0.0013 (5)	−0.0053 (6)	−0.0050 (6)
C24	0.0210 (7)	0.0327 (8)	0.0167 (7)	0.0006 (6)	−0.0090 (6)	−0.0031 (6)
C21	0.0190 (7)	0.0205 (7)	0.0176 (7)	−0.0013 (5)	−0.0068 (5)	−0.0056 (5)
C18	0.0431 (10)	0.0297 (9)	0.0182 (7)	−0.0068 (7)	−0.0091 (7)	−0.0037 (6)
C12	0.0211 (7)	0.0199 (7)	0.0235 (7)	−0.0006 (6)	−0.0102 (6)	−0.0041 (6)
C25	0.0210 (7)	0.0185 (7)	0.0249 (7)	0.0009 (5)	−0.0090 (6)	−0.0045 (6)
C4	0.0269 (8)	0.0220 (8)	0.0219 (7)	0.0033 (6)	−0.0038 (6)	−0.0022 (6)
C23	0.0421 (10)	0.0170 (8)	0.0257 (8)	−0.0006 (7)	−0.0024 (7)	−0.0003 (6)
C10	0.0202 (8)	0.0378 (10)	0.0303 (9)	−0.0047 (7)	−0.0060 (6)	−0.0111 (7)
C7	0.0259 (8)	0.0154 (7)	0.0205 (7)	−0.0001 (5)	−0.0110 (6)	−0.0041 (5)
C17	0.0505 (12)	0.0348 (10)	0.0198 (8)	−0.0098 (8)	−0.0043 (8)	−0.0015 (7)
C13	0.0314 (9)	0.0281 (9)	0.0316 (9)	0.0054 (7)	−0.0085 (7)	−0.0035 (7)
C16	0.0465 (11)	0.0255 (9)	0.0272 (9)	−0.0110 (8)	−0.0048 (8)	0.0004 (7)
C26	0.0275 (10)	0.0493 (12)	0.0578 (13)	0.0141 (9)	−0.0168 (9)	−0.0134 (10)

### Geometric parameters (Å, °)

**Table:**

S1—O2	1.4347 (12)	C14—C15	1.391 (2)
S1—O3	1.4365 (12)	C9—O4	1.2226 (19)
S1—N1	1.6449 (13)	C9—C8	1.462 (2)
S1—C1	1.7548 (16)	C8—C7	1.371 (2)
S2—O9	1.4308 (13)	C20—O8	1.3413 (18)
S2—O10	1.4337 (14)	C20—C21	1.369 (2)
S2—N2	1.6477 (14)	C6—C1	1.401 (2)
S2—C14	1.7504 (16)	C6—C5	1.403 (2)
O8—C20	1.3413 (18)	C6—C7	1.461 (2)
O8—H8O	0.98 (3)	C1—C2	1.391 (2)
O1—C7	1.3377 (18)	C2—H2	0.9500
O1—H1O	0.79 (3)	C15—C16	1.395 (2)
O12—C22	1.3285 (19)	C15—H15	0.9500
O12—C23	1.457 (2)	C5—C4	1.389 (2)
O4—C9	1.2226 (19)	C5—H5	0.9500
O7—C12	1.3330 (19)	C22—C21	1.464 (2)
O7—C13	1.461 (2)	C24—C25	1.510 (2)
O11—C22	1.2329 (19)	C24—H24A	0.9900
O14—C25	1.3423 (19)	C24—H24B	0.9900
O14—C26	1.443 (2)	C18—C17	1.391 (3)
N1—C8	1.4362 (18)	C18—H18	0.9500
N1—C11	1.4658 (19)	C4—H4	0.9500
O13—C25	1.204 (2)	C23—H23A	0.9800
O5—C9	1.3364 (19)	C23—H23B	0.9800
O5—C10	1.4535 (19)	C23—H23C	0.9800
N2—C21	1.4298 (18)	C10—H10A	0.9800
N2—C24	1.4543 (19)	C10—H10B	0.9800
O6—C12	1.204 (2)	C10—H10C	0.9800
C19—C18	1.402 (2)	C17—C16	1.392 (3)
C19—C14	1.403 (2)	C17—H17	0.9500
C19—C20	1.468 (2)	C13—H13A	0.9800
C11—C12	1.521 (2)	C13—H13B	0.9800
C11—H11A	0.9900	C13—H13C	0.9800
C11—H11B	0.9900	C16—H16	0.9500
C3—C4	1.393 (2)	C26—H26A	0.9800
C3—C2	1.393 (2)	C26—H26B	0.9800
C3—H3	0.9500	C26—H26C	0.9800
			
O2—S1—O3	118.97 (7)	C14—C15—H15	120.8
O2—S1—N1	107.65 (7)	C16—C15—H15	120.8
O3—S1—N1	107.63 (7)	C4—C5—C6	120.16 (15)
O2—S1—C1	110.07 (7)	C4—C5—H5	119.9
O3—S1—C1	108.17 (7)	C6—C5—H5	119.9
N1—S1—C1	103.18 (7)	O11—C22—O12	122.69 (14)
O9—S2—O10	119.43 (9)	O11—C22—C21	122.86 (14)
O9—S2—N2	107.81 (8)	O12—C22—C21	114.45 (13)
O10—S2—N2	107.01 (7)	N2—C24—C25	114.97 (13)
O9—S2—C14	110.62 (8)	N2—C24—H24A	108.5
O10—S2—C14	108.43 (8)	C25—C24—H24A	108.5
N2—S2—C14	102.09 (7)	N2—C24—H24B	108.5
C20—O8—H8O	106.3 (17)	C25—C24—H24B	108.5
C7—O1—H1O	107 (2)	H24A—C24—H24B	107.5
C22—O12—C23	115.47 (13)	C20—C21—N2	120.96 (13)
C12—O7—C13	113.54 (13)	C20—C21—C22	119.85 (13)
C25—O14—C26	115.71 (15)	N2—C21—C22	119.16 (13)
C8—N1—C11	118.88 (12)	C17—C18—C19	119.55 (16)
C8—N1—S1	114.54 (10)	C17—C18—H18	120.2
C11—N1—S1	119.24 (10)	C19—C18—H18	120.2
C9—O5—C10	115.65 (12)	O6—C12—O7	124.32 (15)
C21—N2—C24	119.88 (13)	O6—C12—C11	124.73 (14)
C21—N2—S2	114.97 (10)	O7—C12—C11	110.94 (13)
C24—N2—S2	119.48 (11)	O13—C25—O14	125.30 (15)
C18—C19—C14	118.56 (15)	O13—C25—C24	126.78 (14)
C18—C19—C20	121.16 (14)	O14—C25—C24	107.92 (13)
C14—C19—C20	119.98 (14)	C5—C4—C3	120.59 (15)
N1—C11—C12	113.26 (13)	C5—C4—H4	119.7
N1—C11—H11A	108.9	C3—C4—H4	119.7
C12—C11—H11A	108.9	O12—C23—H23A	109.5
N1—C11—H11B	108.9	O12—C23—H23B	109.5
C12—C11—H11B	108.9	H23A—C23—H23B	109.5
H11A—C11—H11B	107.7	O12—C23—H23C	109.5
C4—C3—C2	120.26 (15)	H23A—C23—H23C	109.5
C4—C3—H3	119.9	H23B—C23—H23C	109.5
C2—C3—H3	119.9	O5—C10—H10A	109.5
C15—C14—C19	122.05 (15)	O5—C10—H10B	109.5
C15—C14—S2	121.66 (12)	H10A—C10—H10B	109.5
C19—C14—S2	116.28 (12)	O5—C10—H10C	109.5
O4—C9—O5	123.11 (14)	H10A—C10—H10C	109.5
O4—C9—O5	123.11 (14)	H10B—C10—H10C	109.5
O4—C9—C8	122.76 (14)	O1—C7—C8	123.00 (14)
O4—C9—C8	122.76 (14)	O1—C7—C6	113.63 (13)
O5—C9—C8	114.13 (13)	C8—C7—C6	123.36 (14)
C7—C8—N1	121.26 (14)	C18—C17—C16	121.10 (17)
C7—C8—C9	119.61 (13)	C18—C17—H17	119.4
N1—C8—C9	119.10 (13)	C16—C17—H17	119.4
O8—C20—C21	121.98 (14)	O7—C13—H13A	109.5
O8—C20—C21	121.98 (14)	O7—C13—H13B	109.5
O8—C20—C19	114.68 (13)	H13A—C13—H13B	109.5
O8—C20—C19	114.68 (13)	O7—C13—H13C	109.5
C21—C20—C19	123.29 (14)	H13A—C13—H13C	109.5
C1—C6—C5	118.23 (14)	H13B—C13—H13C	109.5
C1—C6—C7	120.43 (14)	C17—C16—C15	120.20 (17)
C5—C6—C7	121.30 (14)	C17—C16—H16	119.9
C2—C1—C6	121.94 (14)	C15—C16—H16	119.9
C2—C1—S1	120.99 (12)	O14—C26—H26A	109.5
C6—C1—S1	117.05 (12)	O14—C26—H26B	109.5
C1—C2—C3	118.78 (15)	H26A—C26—H26B	109.5
C1—C2—H2	120.6	O14—C26—H26C	109.5
C3—C2—H2	120.6	H26A—C26—H26C	109.5
C14—C15—C16	118.48 (16)	H26B—C26—H26C	109.5
			
O2—S1—N1—C8	165.62 (10)	N1—S1—C1—C2	145.82 (13)
O3—S1—N1—C8	−65.00 (12)	O2—S1—C1—C6	−150.26 (12)
C1—S1—N1—C8	49.24 (12)	O3—S1—C1—C6	78.24 (13)
O2—S1—N1—C11	15.84 (13)	N1—S1—C1—C6	−35.61 (13)
O3—S1—N1—C11	145.22 (11)	C6—C1—C2—C3	−1.9 (2)
C1—S1—N1—C11	−100.54 (12)	S1—C1—C2—C3	176.63 (12)
O9—S2—N2—C21	−167.55 (11)	C4—C3—C2—C1	0.5 (2)
O10—S2—N2—C21	62.81 (12)	C19—C14—C15—C16	1.7 (3)
C14—S2—N2—C21	−50.99 (12)	S2—C14—C15—C16	−176.97 (15)
O9—S2—N2—C24	−14.11 (14)	C1—C6—C5—C4	−0.4 (2)
O10—S2—N2—C24	−143.74 (12)	C7—C6—C5—C4	177.35 (14)
C14—S2—N2—C24	102.45 (12)	C23—O12—C22—O11	5.7 (2)
C8—N1—C11—C12	−70.27 (17)	C23—O12—C22—C21	−175.20 (14)
S1—N1—C11—C12	78.20 (15)	C21—N2—C24—C25	69.53 (18)
C18—C19—C14—C15	−2.8 (3)	S2—N2—C24—C25	−82.61 (16)
C20—C19—C14—C15	170.92 (15)	O8—C20—C21—N2	−175.35 (14)
C18—C19—C14—S2	175.91 (13)	O8—C20—C21—N2	−175.35 (14)
C20—C19—C14—S2	−10.3 (2)	C19—C20—C21—N2	7.1 (2)
O9—S2—C14—C15	−25.92 (17)	O8—C20—C21—C22	2.7 (2)
O10—S2—C14—C15	106.82 (15)	O8—C20—C21—C22	2.7 (2)
N2—S2—C14—C15	−140.43 (14)	C19—C20—C21—C22	−174.91 (14)
O9—S2—C14—C19	155.31 (13)	C24—N2—C21—C20	−121.58 (16)
O10—S2—C14—C19	−71.94 (14)	S2—N2—C21—C20	31.75 (19)
N2—S2—C14—C19	40.81 (14)	C24—N2—C21—C22	60.39 (19)
O4—O4—C9—O5	0.00 (13)	S2—N2—C21—C22	−146.28 (12)
O4—O4—C9—C8	0.00 (6)	O11—C22—C21—C20	4.7 (2)
C10—O5—C9—O4	−1.3 (2)	O12—C22—C21—C20	−174.38 (14)
C10—O5—C9—O4	−1.3 (2)	O11—C22—C21—N2	−177.28 (14)
C10—O5—C9—C8	178.51 (14)	O12—C22—C21—N2	3.7 (2)
C11—N1—C8—C7	114.97 (16)	C14—C19—C18—C17	1.7 (3)
S1—N1—C8—C7	−34.92 (18)	C20—C19—C18—C17	−171.98 (18)
C11—N1—C8—C9	−66.85 (18)	C13—O7—C12—O6	5.0 (2)
S1—N1—C8—C9	143.26 (12)	C13—O7—C12—C11	−174.02 (13)
O4—C9—C8—C7	−6.3 (2)	N1—C11—C12—O6	7.2 (2)
O4—C9—C8—C7	−6.3 (2)	N1—C11—C12—O7	−173.69 (12)
O5—C9—C8—C7	173.89 (13)	C26—O14—C25—O13	−2.1 (2)
O4—C9—C8—N1	175.49 (14)	C26—O14—C25—C24	178.36 (15)
O4—C9—C8—N1	175.49 (14)	N2—C24—C25—O13	−0.1 (2)
O5—C9—C8—N1	−4.3 (2)	N2—C24—C25—O14	179.43 (13)
O8—O8—C20—C21	0.00 (15)	C6—C5—C4—C3	−1.0 (2)
O8—O8—C20—C19	0.00 (19)	C2—C3—C4—C5	1.0 (2)
C18—C19—C20—O8	−22.0 (2)	N1—C8—C7—O1	178.77 (13)
C14—C19—C20—O8	164.35 (14)	C9—C8—C7—O1	0.6 (2)
C18—C19—C20—O8	−22.0 (2)	N1—C8—C7—C6	−2.2 (2)
C14—C19—C20—O8	164.35 (14)	C9—C8—C7—C6	179.64 (14)
C18—C19—C20—C21	155.70 (17)	C1—C6—C7—O1	−163.40 (14)
C14—C19—C20—C21	−17.9 (2)	C5—C6—C7—O1	18.9 (2)
C5—C6—C1—C2	1.8 (2)	C1—C6—C7—C8	17.5 (2)
C7—C6—C1—C2	−175.91 (14)	C5—C6—C7—C8	−160.19 (15)
C5—C6—C1—S1	−176.74 (11)	C19—C18—C17—C16	0.5 (3)
C7—C6—C1—S1	5.53 (19)	C18—C17—C16—C15	−1.6 (3)
O2—S1—C1—C2	31.17 (15)	C14—C15—C16—C17	0.5 (3)
O3—S1—C1—C2	−100.33 (14)		

### Hydrogen-bond geometry (Å, °)

**Table:**

*D*—H···*A*	*D*—H	H···*A*	*D*···*A*	*D*—H···*A*
O1—H1O···O4	0.79 (3)	1.87 (3)	2.5754 (18)	149 (3)
O8—H8O···O11	0.98 (3)	1.68 (3)	2.5623 (17)	148 (3)
C13—H13A···O10^i^	0.98	2.44	3.202 (2)	134.
C2—H2···O11^ii^	0.95	2.59	3.339 (2)	136.

Symmetry codes: (i) −*x*, −*y*+2, −*z*+1; (ii) −*x*, −*y*+1, −*z*+1.
